# Increasing Higher Alcohols and Acetates in Low-Alcohol Beer by Proteases

**DOI:** 10.3390/molecules28114419

**Published:** 2023-05-29

**Authors:** Claire Lin Lin, Mikael Agerlin Petersen, Andrea Gottlieb

**Affiliations:** 1 Brewing AR 345, Novozymes A/S, Biologiensvej 2, 2800 Kongens Lyngby, Denmark; 2 Department of Food Science, University of Copenhagen, Rolighedsvej 26, 1958 Frederiksberg, Denmark

**Keywords:** proteases, non-conventional yeasts, low-alcohol beer, design of experiments, Ehrlich pathway

## Abstract

The market of non-alcoholic and low-alcohol beer has grown continuously thanks to the advocacy for healthy and responsible drinking. Non-alcoholic and low-alcohol products usually possess less higher alcohols and acetates and more aldehyde off-flavors due to the manufacturing processes. The employment of non-conventional yeasts partially mitigates this problem. In this study, we used proteases to optimize the wort amino acid profile for better aroma production during yeast fermentation. The design of experiments was applied to increase the leucine molar fraction, aiming to boost 3-methylbutan-1-ol and 3-methylbutyl acetate (banana-like aromas). This led to an increase from 7% to 11% leucine in wort after protease treatment. The aroma output in the subsequent fermentation, however, was yeast-dependent. An 87% increase of 3-methylbutan-1-ol and a 64% increase of 3-methylbutyl acetate were observed when *Saccharomycodes ludwigii* was used. When *Pichia kluyveri* was employed, higher alcohols and esters from valine and isoleucine were increased: 58% more of 2-methylpropyl acetate, 67% more of 2-methylbutan-1-ol, and 24% more of 2-methylbutyl acetate were observed. Conversely, 3-methylbutan-1-ol decreased by 58% and 3-methylbutyl acetate largely remained the same. Apart from these, the amounts of aldehyde intermediates were increased to a varying extent. The impact of such increases in aromas and off-flavors on the perception of low-alcohol beer remains to be evaluated by sensory analysis in future studies.

## 1. Introduction

The global market of non-alcoholic and low-alcohol beer (NABLAB) has greatly expanded in past decades due to an increasing consumer concern about health and alcohol consumption [1]. Two types of methods are currently used in NABLAB production: physical methods resulting in de-alcoholization and biological methods involving interrupted fermentation or the application of non-conventional yeasts [2,3,4]. Physical methods entail alcohol evaporation and membrane filtration [2,4]. Both processes risk the loss of aroma compounds and render a product less resemblant to regular beer [5,6,7]. Meanwhile, a biological method employing non-conventional yeasts has flourished with strain and aroma diversity [8,9,10,11]. These yeasts oftentimes lack the ability to ferment maltose, the most abundant fermentable sugar in wort, and thus produce less alcohol compared to conventional *Saccharomyces* yeasts [12,13,14].

NABLAB is usually associated with a worty off-flavor and lack of mouthfeel or taste compared to its regular beer counterpart. In a comparison study, NABLAB was shown to be significantly deficient in the key beer aromas when compared to regular beer [7,15]. These aromas included 2-methylbutan-1-ol, 3-methylbutan-1-ol, ethyl acetate, ethyl hexanoate, and ethyl octanoate [16]. In the same study, the sensory evaluation of NABLAB was associated with “worty”, “watery”, and “sugary”; whereas regular beer scored higher in bitterness and fullness (mouthfeel). Interestingly, this study also documented the different amino acid profiles in NABLAB and in the corresponding regular beer. In particular, NABLAB showed deficiencies in glutamine and alanine [16].

The employment of proteases can change the amino acid profile of wort and potentially impact the aroma profile of the resulting beer via the Ehrlich metabolic pathway in yeasts. In modern brewing practice, proteases have been used to enrich wort free amino nitrogen to compensate for poor malt quality or a high proportion of non-malt adjuncts in the grain mix. The utilization of proteases has been shown to improve yeast performance in high-gravity brewing [17] and to enhance aroma output in normal-gravity brewing [18]. Compared to amino acid spiking experiments [19], applying proteases to generate leucine, isoleucine, valine, and phenylalanine in situ increased the amount of these amino acids without dramatically altering their molar fractions [20]. This limited the aroma effects of these amino acids on downstream fermentation by yeasts.

To maximize the amino acid molar fraction by this protease method, statistically designed experiments were implemented. The design of experiments refers to process planning to statistically collect and analyze data, maximizing the information retrieved from a limited number of experiments [21]. This method has been efficiently applied to optimize processes in industry [22,23]. Several examples have demonstrated its use in food and beverage processes and product development [24,25,26]. Given the extent of the aroma impact among the four chosen amino acid precursors for the Ehrlich pathway [16], leucine was selected as the target. In the current study, there is an interest to understand how several variables (endoprotease, exoprotease, and temperature) plus the interactions among these variables affect the outcome of the leucine molar fraction in wort. This would lead to a more efficient protease usage and potentially a more flavorsome fermentation by yeasts.

In this work, we used the design of experiments to optimize a protease method leading to higher leucine molar fractions. The best combinations of endo- and exoproteases from previous work [20] were employed. An increase from 7% to 11% leucine was obtained after applying proteases in mashing. The aroma production in subsequent fermentation to make low-alcohol beer was yeast-dependent. Fermentation of protease-treated wort was carried out by two commercially important non-conventional yeasts: *Saccharomycodes ludwigii* and *Pichia kluyveri*. An 87% increase of 3-methylbutan-1-ol and a 64% increase of 3-methylbutyl acetate were achieved when using *Saccharomycodes ludwigii*. When *Pichia kluyveri* was applied, however, 3-methylbutan-1-ol decreased by 58% and 3-methylbutyl acetate largely remained the same. The extra amount of leucine present in wort affected higher alcohols and esters from valine and isoleucine, the other two branched-chain amino acids. In the case of *Saccharomycodes ludwigii*, 51% more of 2-methylbutyl acetate from isoleucine was observed. For *Pichia kluyveri*, 58% more of 2-methylpropyl acetate from valine, 67% more of 2-methylbutan-1-ol, and 24% more of 2-methylbutyl acetate from isoleucine were obtained. In addition, the aroma output of these yeasts was compared to a conventional lager yeast, *Saccharomyces pastorianus*, in a 4-day fermentation process.

## 2. Results and Discussion

### 2.1. Design of Experiments and Mashing with Proteases

#### 2.1.1. Design of Experiments to Maximize the Leucine Molar Fraction in Wort

##### Effect Screening of Endoprotease, Exoprotease, and Temperature

Previously, the fermentation of wort derived from protease treatment showed that the amino acid molar fraction plays a more important role in downstream aroma generation compared to its concentration [20]. To maximize the leucine molar fraction by proteases, the design of experiments was used in this work to study the main effects from endoprotease, exoprotease, and temperature. In addition, second-order interactions such as endoprotease × exoprotease, endoprotease × temperature, exoprotease × temperature, and third-order interaction endoprotease × exoprotease × temperature were studied to maximize the leucine molar fraction in protease-treated mashing. Because of the interest in the specific interactions mentioned above, a computer-generated screening design (i.e., custom screening design) was applied to allow maximal flexibility with a low experimental budget. Based on our former study [20], two optimal protease combinations to generate leucine were used to test this method: Neutrase^®^ 0.8 L BrewQ (Endo1) + Protana^®^ Prime (Exo1) and Endo1 + Exo3, an experimental protease identified in SEQ ID NO. 79924 of WO2006069610 [27].

The screening design was generated by JMP^®^ Pro 16.2.0 using *D-*optimal design. There were three continuous factors with two levels; endoprotease dosage and exoprotease dosage ranged from 0 to 100 mg EP/kg-grist, and temperature ranged from 52 to 63 °C. The upper limit of enzyme concentration was restricted by the materials available. The temperature limit was set by two criteria: (1) industrial relevance and (2) endoprotease thermostability because endoprotease had a higher impact on amino acid release than exoprotease [20]. A total of 12 runs including duplicates of four conditions was recommended by JMP, tabulated in Table 1. Replicates were used to estimate experimental errors for statistical testing.

**Figure 1:**
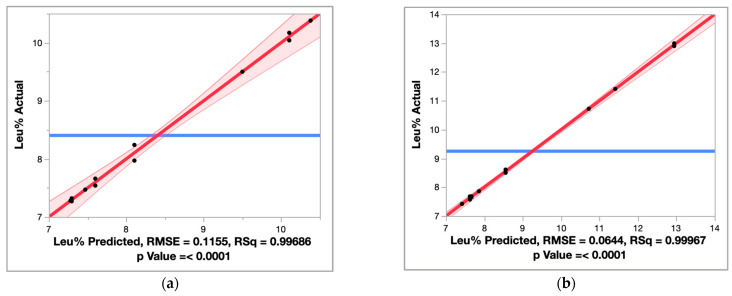
The actual by predicted plot of (**a**) Endo1 + Exo1; (**b**) Endo1 + Exo3. The actual by predicted plot is a scatter plot, with the predicted values as the *x*-axis and the actual values as the *y*-axis. The blue line represented the null hypothesis, wherein only an intercept was plotted without considering any factors. In this case, the null hypothesis means that the varied Leu% was independent of any factors listed in Table 2 as a consequence of mere chance. When these factors were added into the intercept, the red line was generated. A faded shade surrounding the red line represents the 95% confidence interval region. If the blue line fell into the 95% confidence interval region, it would signify that the model test would not be significant; the factors in Table 2 would not account for the observed effect on Leu%. Both (**a**,**b**) show that these factors of interest indeed affected Leu% and these influences were significant, testified by the reported *p* values.

Effect screening was used to identify the active factors among the specified interactions mentioned in the statistical model. The two “actual by predicted” plots of Endo1 + Exo1 and Endo1 + Endo3 (Figure 1) indicated no obvious lack of fit. The models built with the main effects and specified interaction terms explained 99.69% variance (Endo1 + Exo1) and 99.97% variance (Endo1 + Exo3) of the leucine molar fraction. The effect test showed that all seven model terms were significant (*p* ≤ 0.05, Table 2).

**Figure A1:**
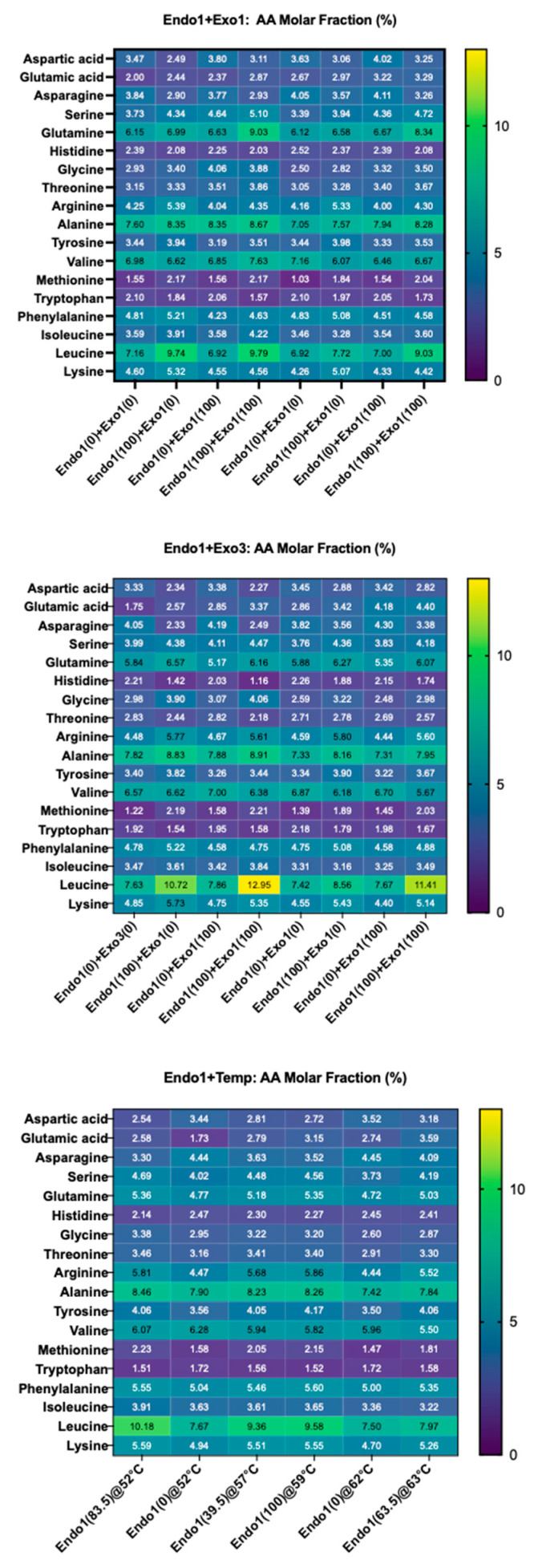
Wort amino acid molar fraction in mashing trials for design of experiments (Section 2.1.1). Numbers in parentheses in the row labels indicate enzyme dosages used, in unit of mg EP/kg-grist. Color mapping of amino acid molar fractions range from 0% to 13%.

**Figure A2:**
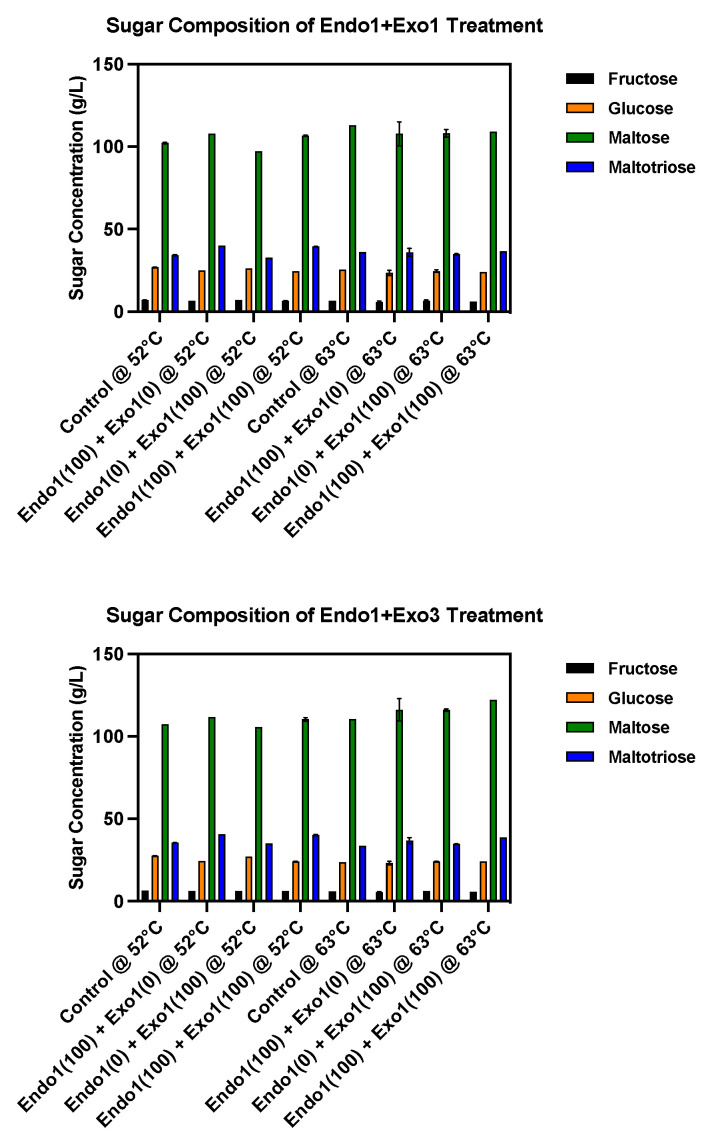
Wort (approximately 21 °P) sugar composition including fructose, glucose, and maltose after enzyme treatments. Numbers in parentheses indicate enzyme dosages used in unit of mg EP/kg-grist.

The estimated parameters and the effect summaries in Table 2 for the two enzyme treatments suggest different interactions in Endo1 + Exo1 and Endo1 + Exo3. In both cases, the endoprotease Endo1 had a positive and major impact on leucine generation, demonstrated by its positive parameters in both enzyme combinations. Since endoprotease was thermal labile, temperature had a negative influence on the leucine response—negative parameters in both Endo1 + Exo1 and Endo1 + Exo3—due to the reduced enzyme activity at elevated temperatures. Differences brought by the two exoproteases, Exo1 and Exo3, affected interaction terms such as endoprotease × exoprotease, exoprotease × temperature, and endoprotease × exoprotease × temperature. When the temperature impact was further inspected, parameter values for the second-order Exo1 × Temp and the third-order Endo1 × Exo1 × Temp were larger than those for Exo3 × Temp and Endo1 × Exo3 × Temp, respectively. This may imply that temperature had a bigger influence on the activity of Exo1 and the interaction of Endo1 and Exo1 compared to those of Exo3. Moreover, when comparing the exoproteases, the parameter values for the main effect of Exo3 and the interaction term Endo1 × Exo3 were larger than those for Exo1 and Endo1 × Exo1, respectively. This suggests a more favorable influence on the leucine molar fraction from Exo3 than that from Exo1. Overall, the main effects from endoprotease and exoprotease seem larger in the Endo1 + Exo3 combination based on the estimated parameters. Additionally, Endo1 + Exo3 did not increase the glutamate molar fraction (Appendix A, Figure A1). Since glutamate was the preferred nitrogen source compared to leucine, the presence of glutamate could slow down leucine uptake via the nitrogen catabolite repression mechanism [28,29,30]. Based on these rationales, Endo1 + Exo3 was chosen as the protease combination in mashing to produce wort for downstream fermentation. Neither Endo1 + Exo1 nor Endo1 + Exo3 affected the wort sugar profile (Appendix A, Figure A2).

##### Response Surface Model of Endoprotease Endo1 and Temperature

Although it is the main variable affecting leucine production, Endo1 is not thermally stable; hence, its activity is influenced by temperature. Therefore, a surface response model was set up to study the interaction between Endo1 and temperature. The quadratic term endoprotease × endoprotease was assumed based on saturation kinetics. The effect from Endo1 is expected to reach a plateau when the substrate is depleted in wort, and no product (leucine) increase would be observed despite the increasing amount of endoprotease. The quadratic term temperature × temperature describes the rate curvature of a general enzymatic reaction. Higher temperatures can enhance the reaction rate, but temperatures higher than the enzyme’s optimal temperature can also destabilize the enzyme and reduce its activity. This eventually leads to denaturation. In addition, the interaction term endoprotease × temperature accounts for the thermal liability of Endo1: Endo1 activity decreases as temperature increases above its optimal temperature. To allow flexibility, a custom design was applied with response surface modeling settings. A total of 8 runs covering endoprotease dosages from 0 to 100 mg EP/kg-grist and temperatures from 52 to 63 °C were generated by JMP, tabulated in Table 3. The Endo1 dosage could easily be manipulated, yet the temperature in the lab-scale mashing device could only be adjusted to the nearest unit °C. Therefore, Endo1 was set as a continuous variable, and temperature was set as a discrete numeric variable.

**Figure 2:**
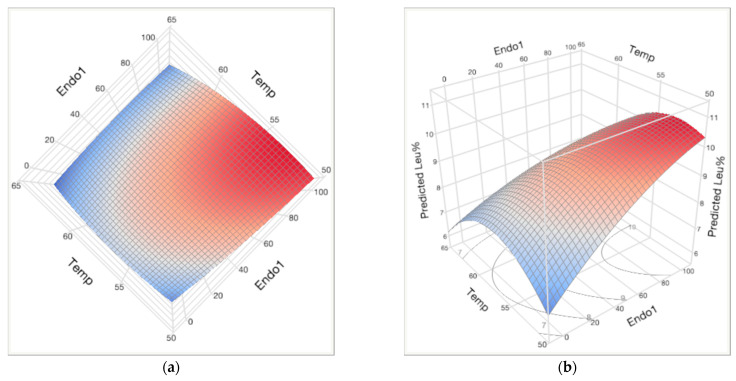
Response surface predicted by JMP: (**a**) top view of the response surface (red designates high Leu% and blue designates low Leu%); (**b**) profile view of the response surface with contour underneath: Leu% values from 7% to 10% are labelled.

The model generated (Table 4) suggested that both main effects, Endo1 and temperature, impacted the outcome significantly. Temperature negatively influenced the outcome due to the destabilization of endoprotease at higher temperatures. This explained the negative coefficients of all second-order terms in the model (Figure 2). These coefficients revealed the influence of these second-order terms on the response: Temp × Temp having the most impact, implicating that the response would change fast when temperature changed, followed by the interaction of temperature and Endo1, and finally Endo1 × Endo1. These effects were illustrated by the curvature of the response surface in the intersections of Endo1 versus response and that of temperature versus response, respectively (Figure 2b).

**Figure 3:**
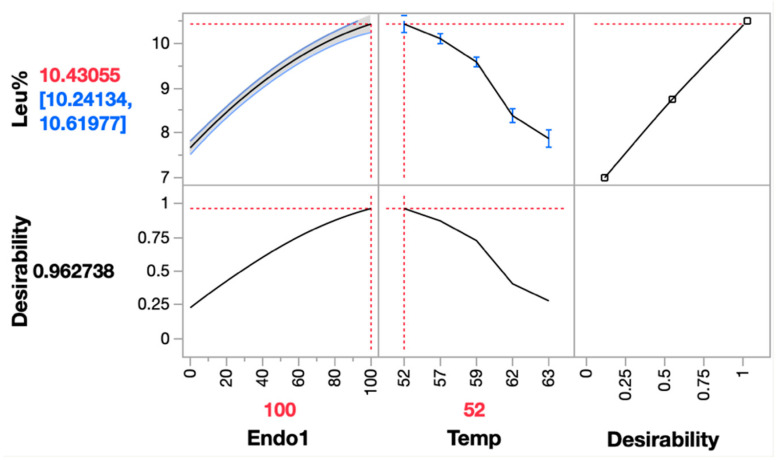
Prediction profiler of the response surface of Leu% with maximal desirability. Optimal condition was chosen as Endo1 at 100 mg EP/kg-grist and temperature at 52 °C, highlighted in red. The predicted Leu% would be 10.43055% (in red) under these conditions, locating within the confidence interval from 10.24134% to 10.61977% (in blue).

The optimal temperature (peak of the parabola) at each endoprotease dosage approximately overlapped with the long axis of the incomplete ellipse in Figure 2a. Following this axis, the predicted maximum was outside of the region at approximately 106 mg EP/kg-grist. Within the region of allowed enzyme dosages and temperatures, 100 mg EP/kg-grist at 52 °C was chosen as the optimal condition to maximize the leucine molar fraction output (Figure 3).

In summary, the design of experiments was used to choose the optimal settings to maximize the leucine molar fraction in wort after mashing. This concluded on an experimental condition using Endo1 and Exo3 at 52 °C, both at 100 mg EP/kg-grist.

#### 2.1.2. Fermenting Wort Production and Amino Acid Molar Fraction

**Figure 4:**
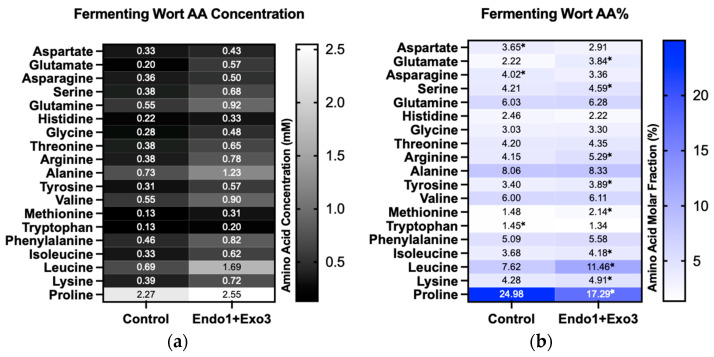
Amino acid profile after mashing: (**a**) amino acid expressed in mM; (**b**) amino acid expressed in molar fraction. Amino acid molar fractions with asterisks designate statistically significant differences (*p* < 0.05).

Based on our results from the effect screening and response-surface modeling, Endo1 at 100 mg/kg-grist, Exo3 at 100 mg/kg, and 52 °C were chosen as the mashing condition. Lab-scale mashing treated by proteases produced about threefold the amount of leucine compared to the control (Figure 4a). Other yeast preferred amino acids [31] that showed a similar level of change, were alanine (twofold), arginine (twofold), glutamate (twofold), glutamine (twofold), and serine (twofold). Increases of these amino acids influence the uptake of less-preferred flavor-active amino acids such as leucine and phenylalanine. Overall, protease treatment increased the total free amino acid concentration from 9 mM to 15 mM.

Despite the increase in amino acid concentration, only the leucine molar fraction changed to a great extent (Figure 4b). The molar fractions of yeast preferred amino acids alanine, arginine, glutamate, glutamine, and serine stayed roughly the same before and after protease treatment. Leucine, however, increased from 7.62% to 11.46%. The proline molar fraction dropped from 24.98% to 17.29% after protease treatment, caused by the increase of other amino acids.

**Figure A3:**
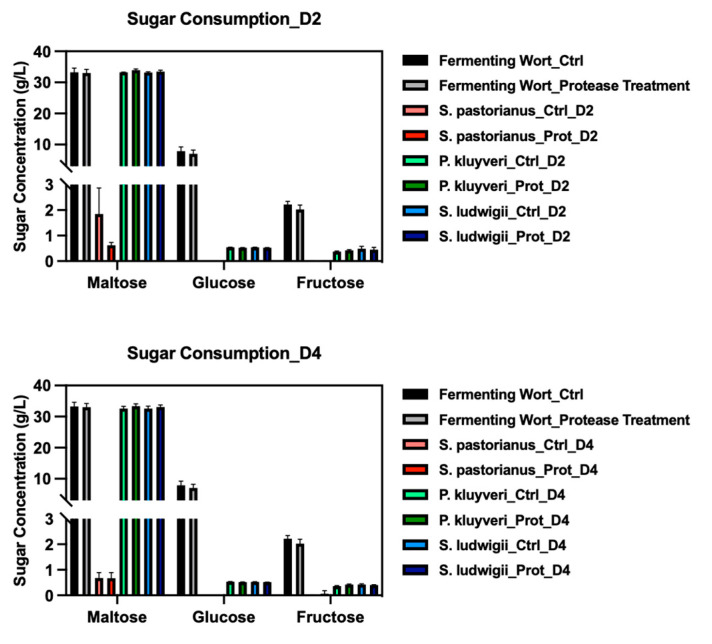
Wort sugar composition including fructose, glucose, and maltose. Sugar consumption on Day 2 (**top**) and Day 4 (**bottom**) fermentation by various yeasts. Fermenting wort was initially 7 °P.

The leucine molar fraction in this experiment only reached 11.46%, different from the expected value of 12.99% achieved in the effect screening trial (Table 1). This may be explained by the applied boiling step between wort production and yeast fermentation. The boiling step involved wort heating in a 100 °C water bath for 60 min. It served the goal of sanitizing the fermenting wort before yeast pitching. However, denatured proteins would precipitate, possibly complexed with some free amino acids (and polyphenols)—the “hot break” in brewing terms. Unfortunately, this process was overlooked both in effect screening and in response surface modeling. This would explain the above observation that only 11.46% of Leu was obtained despite the favorable enzyme dosages and mashing condition. The fermentable sugar composition was not influenced by the protease treatment (Appendix A, Figure A3).

### 2.2. Fermentation and Aroma Output

**Figure 5:**
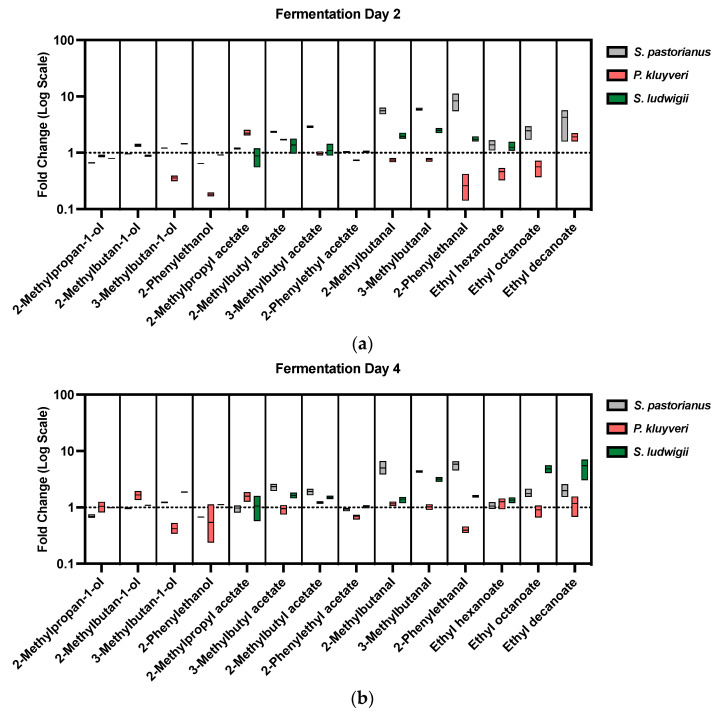
Fold change of volatiles from protease-treated wort compared to those from non-enzyme-treated control after: (**a**) 2-day and (**b**) 4-day fermentation. All fermentations were carried out in triplicate. Volatile compounds from every type of yeast in protease-treated wort were referenced to the same type of yeast in the control wort. Values are represented as floating columns stretching from the minimum to the maximum, plotted in a base 10 logarithmic scale; the middle bar designates the mean. For each volatile, values are arranged and colored according to the yeasts: **left**, *S. pastorianus*, in grey; middle, *P. kluyveri*, in red; **right**, *S. ludwigii*, in green. Undetected volatiles (ethyl octanoate and ethyl decanoate in Day 2 fermentation by *S. ludwigii*) are shown as missing columns. A reference ratio of 1 is shown in a dotted grey line.

Fermenting wort derived from protease-treated mash was subsequently fermented by Weihenstephan 34/70 (WS 34/70), a *Saccharomyces pastorianus* strain, (*S. pastorianus*), *Pichia kluyveri* (*P. kluyveri*), and *Saccharomycodes ludwigii* (*S. ludwigii*). *S. ludwigii* and *P. kluyveri* are commercially important because of their application in low-alcohol and non-alcoholic beer production [1,13]. *S. ludwigii* has been used to make low-alcohol and non-alcoholic beer due to its inability to ferment maltose, the major sugar component in wort. *P. kluyveri* has recently gained popularity thanks to its superior ability to produce fruity aromas, adding a pleasant impression to the otherwise plain aroma profile of low-alcohol beer [32]. Fermentation was compared among *S. ludwigii*, *P. kluyveri*, and WS 34/70 under two conditions: non-enzyme-treated control and enzyme-treated wort. The attenuation and alcohol content in the resulting beer are tabulated in Table A1 (Appendix A). The aroma output is summarized in Figure 5b and Table A2 (Appendix A).

**Figure 6:**
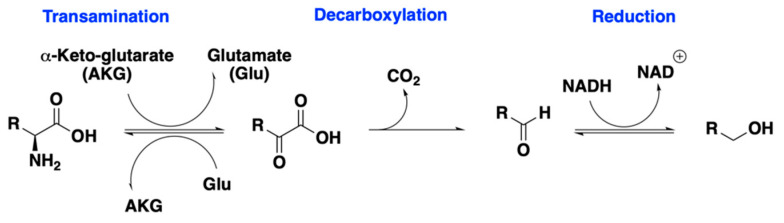
General scheme of the Ehrlich pathway: amino acid catabolism leading to higher alcohols. Adapted with permission from Ref. [42]. Copyright © 2014, Springer-Verlag Berlin Heidelberg.

**Figure 7:**
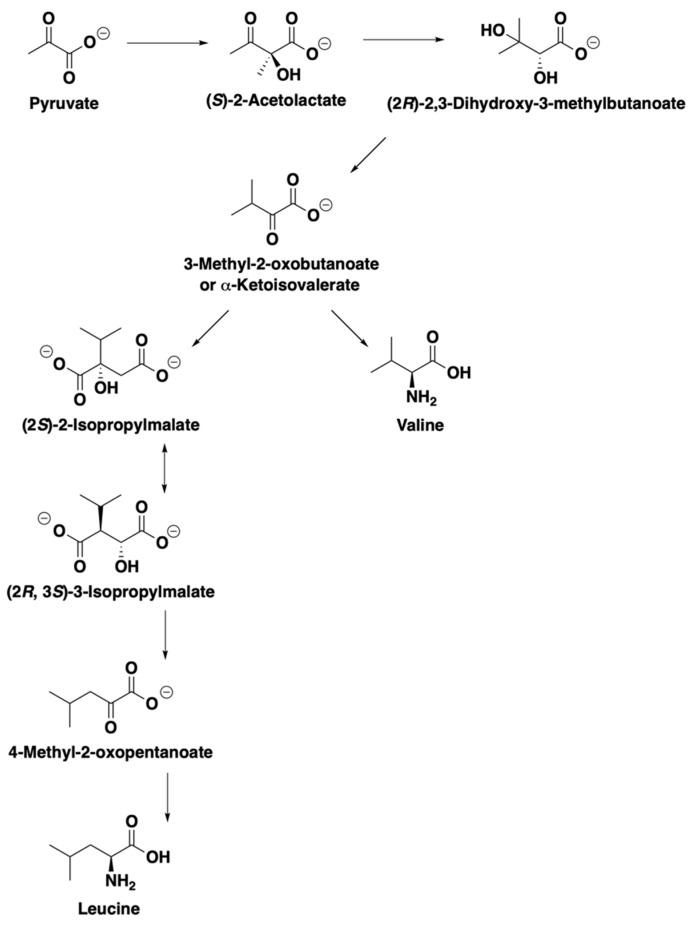
Yeast biosynthesis of leucine and valine connected by a common intermediate: α-ketoisovalerate. Adapted from the *Saccharomyces* Genome Database (SGD), Refs. [36,37,38,39,40]. SGD content is licensed under the Creative Commons Attribution 4.0 International license, available at https://creativecommons.org/licenses/by/4.0/.

Wort derived from protease treatment led to changes of a few key aromas and aldehyde off-flavors. For *P. kluyveri*, the increase of branched-chain amino acids in wort led to corresponding higher alcohols and acetates: 2-methylpropan-1-ol from valine and 2-methylbutan-1-ol from isoleucine (Figure 6). Although the protease treatment aimed to maximize the leucine molar fraction, the increase of leucine led to a decrease of 3-methylbutan-1-ol and almost an equal amount of 3-methylbutyl acetate. The extra leucine might interfere in the yeast uptake of isoleucine [33,34,35], as evidenced by the evolution of 2-methylbutanal, 2-methylbutan-1-ol, and 2-methylbutyl acetate on Day 2 and Day 4 (Figure 5a,b, Tables S1 and S2). Meanwhile, the additional leucine might be directed to valine metabolism via a common intermediate, α-ketoisovalerate [36,37,38,39,40] (Figure 7). This could also be seen from the evolution patterns of 2-methylpropan-1-ol and 2-methylpropyl acetate (Figure 5a,b, Tables S1 and S2). Interestingly, the reoccurring trend in *Saccharomyces* yeasts that increasing leucine decreases phenylalanine-derived aromas [20,31,41] was observed in both *S. pastorianus* and *P. kluyveri*. Both 2-phenylethanol and 2-phenylethyl acetate concentrations were lower after protease treatment compared to the control in fermentation employing *P. kluyveri* (Figure 5b, Table A2). Fermentation using *S. pastorianus* showed a decrease in 2-phenylethanol and a slight decrease in 2-phenylethyl acetate production (Figure 5b, Table A2). On the other hand, *S. ludwigii* showed an increase in 3-methylbutan-1-ol and 3-methylbutyl acetate production from leucine generated in situ by proteases. Different than *P. kluyveri* and *S. pastorianus*, *S. ludwigii* showed slight increases in 2-phenylethanol and 2-phenylethyl acetate despite the presence of more leucine after protease treatment, suggesting different amino acid metabolism than the other two yeasts. In addition, protease treatment led to the increase of medium-chain fatty acid ethyl esters, particularly in fermentation performed by *S. ludwigii* (Figure 5b).

In addition to higher alcohols and acetates, their corresponding aldehyde intermediates were also higher due to the increase of these branched-chain amino acids and phenylalanine from protease treatment. In the case of *S. pastorianus*, 3-methylbutanal, 2-methylbutanal, and 2-phenylethanal increased four to fivefold after protease treatment (Figure 5a,b, Table A2). This was due to the increased metabolism of these amino acids in yeasts. The same trend was observed in *S. ludwigii*, albeit with a different extent of increment (Figure 5a,b). However, for *P. kluyveri*, these three aldehydes decreased on Day 2 despite the higher amounts of amino acids present in protease-treated wort (Figure 5a and Table S1). On Day 4, the concentrations of 3-methylbutanal and 2-methylbutanal caught up with those in the control, while 2-phenylethanal remained low in comparison with that in control (Figure 5b and Table S2). The volatile evolution of higher alcohols, acetates, and corresponding aldehydes from leucine and phenylalanine was also studied. While aroma production depended on the strains, the general trend of evolution showed a similar pattern between the two wort conditions (Figure 5a,b).

In summary, protease treatment targeting leucine led to an 87% increase of 3-methylbutan-1-ol and a 64% increase of 3-methylbutyl acetate after 4-day fermentation by *S. ludwigii* (Table A2). When a normal lager yeast, *S. pastorianus,* was used, only a 23% increase of 3-methylbutan-1-ol and almost the same amount of 3-methylbutyl acetate were produced. *P. kluyveri*, on the other hand, generated 58% more 2-methylpropyl acetate, 67% more 2-methylbutan-1-ol, and 24% more 2-methylbutyl acetate versus the control; whereas 3-methylbutan-1-ol decreased by 58%, and 3-methylbutyl acetate largely remained the same. The chemical results obtained by GCMS for *S. ludwigii* showed that protease treatment may have the potential to correct higher alcohol and acetate deficiencies in low-alcohol beer produced by this strain. The same treatment may make the fruity impression of *P. kluyveri* even stronger. However, the actual aroma impression from protease treatment would need sensory analysis for proper evaluation.

## 3. Materials and Methods

### 3.1. Mashing and Wort Production

A comprehensive description of the materials, reagents, instruments, and procedures was documented in previous work [20]. Consequently, only key experimental approaches are described below.

A high-gravity mashing experiment with a liquor-to-grist ratio of 3 was conducted to imitate industrial setup. A total of 75 g of barley malt and 225 mL of deionized water containing 3 mM CaCl_2_ were mixed in a laboratory-scale mashing device LB-12 as previously described [20]. An infusion mashing program was employed: 52 °C for 30 min, 63 °C for 30 min, 72 °C for 20 min, and 78 °C for 10 min. The resulting wort was obtained by filtration as before [20]. Fermentable sugar in the resulting wort such as fructose, glucose, and maltose were measured by high-performance liquid chromatography coupled with a refractive index detector as previous described [20]. Free amino acid measurement was carried out in ultra-performance liquid chromatography coupled with a fluorescence detector [20]. Primary amino acids underwent inline derivatization with *O*-phthaldialdehyde (OPA) and were detected at 338 nm; the secondary amino acid, proline, was coupled with 9-fluorenylmethylchloroformate (FMOC) inline and monitored at 460 nm. An internal standard (norvaline for all but proline, and sarcosine for proline) was applied to construct a standard curve, against which the concentration of individual amino acids was calibrated. The specific gravity (°P) of the resulting wort was measured as before [20]. It was standardized with deionized water to 7 °P before boiling in a 100 °C water bath for 60 min. The boiled wort was filtered via a sterile assembly in laminar flow as before to obtain the fermenting wort [20].

### 3.2. Fermentation and Volatile Measurement

Pitching yeasts were prepared in a modified procedure from a previous study [20]. Three bottles containing 150 mL of 7 °P sterile wort (Section 3.1) were inoculated with three different yeast strains from *S. pastorianus* (commercial lager strain: Saflager W-34/70, Fermentis, France), *P. kluyveri*, and *S. ludwigii* (proprietary strains from Novozymes Central Collection, Kongens Lyngby, Denmark), respectively. *S. pastorianus* (1 g) was added as dry yeast from a package. *P. kluyveri* and *S. ludwigii* were, respectively, propagated from a single colony on a yeast extract-peptone-dextrose (YPD) agar plate. Yeasts were grown in a climate-controlled orbital sharker at 22 °C, 120 rpm for 72 h, labeled as the 1st generation. A portion (5 mL) of this 1st generation preculture was inoculated into another 300 mL sterile wort of 7 °P and incubated for 48 h under the same condition, labeled as the 2nd generation. Yeast cells were concentrated and washed in sterile ultrapure water by centrifugation at 4000× *g* for 5 min. The cell count and yeast viability test (using methylene blue) were carried out following standardized procedures described in former work [20]. *S. pastorianus*, *P. kluyveri*, and *S.ludwigii* were, respectively, reported as 91%, 100%, and 100% viable before pitching.

Fermenting wort of 7 °P obtained in Section 3.1 was distributed in a portion of 75 mL to a 100 mL sterile glass bottle equipped with an airlock. Fermentation trials were carried out in triplicate: three strains were independently used to ferment two different types of wort (protease-treated one versus control). Volatile productions were investigated at two timepoints: Day 2 and Day 4 of fermentation. In summary, a total of 36 fermentation bottles were placed in a climate-controlled orbital sharker at 22 °C, 120 rpm, the same settings as above. Yeasts were pitched at 1.5 × 10^7^ viable cells/mL wort. Each fermentation was terminated upon the set timepoint by slow decantation to separate approximately 30 mL of the green beer from the flocculated yeast cells. Care was taken to avoid the disturbance of the yeast cells settled at the bottom of the fermentation bottle. Volatiles in green beer samples (5 mL) were immediately isolated using the stir bar sorptive extraction method as described in a previous study [20]. The remaining green beer samples were frozen and stored at −18 °C before further analysis.

Volatile measurement was performed on gas chromatography coupled with a single quadrupole mass spectrometer. Detailed instrumentation settings were documented in former work [20]. Volatile extraction and identification were carried out using a software, the Deconvolution and Identification System PARADISe, developed based on PARAFAC2 modeling (University of Copenhagen, Copenhagen, Denmark) [43]. Higher alcohols and acetates derived from leucine, isoleucine, valine, and phenylalanine—3-methylbutan-1-ol, 2-methylbutan-1-ol, 2-methylpropan-1-ol, 2-phenylethanol, and their corresponding acetates—were validated using commercial analytical standards as before [20].

### 3.3. Design of Experiments and Statistical Analysis

A custom effect screening design and a custom response surface modeling were generated with defined dosages and temperatures using JMP^®^ Pro 16.2.0. Amino acid concentrations were statistically evaluated by one-way analysis of variance (ANOVA) followed by Student’s *t*-test. Fermentation aromas were statistically evaluated by two-way ANOVA to account for yeast and wort interaction, followed by Turkey’s test. A confidence interval of 95% was used for pairwise comparison in JMP^®^ Pro 16.2.0.

## 4. Conclusions

In summary, the current study has shown the potential to increase the leucine molar fraction by the design of experiments and the aroma impact of protease treatment on downstream fermentation. While beer aroma profiles are predominantly strain-dependent, protease treatment can increase key aroma compounds such as 2- and 3-methylbutan-1-ol, 2- and 3-methylbutyl acetate, as well as medium-chain fatty acid ethyl esters—ethyl hexanoate, ethyl octanoate, and ethyl decanoate. This has demonstrated the potential of proteases to correct amino acid and aroma deficiencies in non-alcoholic and low-alcohol beer produced by non-conventional yeast strains. However, this protease method can also cause more aldehyde intermediates from the Ehrlich pathway. The true aroma impact remains to be evaluated in future sensory analysis.

## Figures and Tables

**Table 1:** The 12-run *D*-optimal design by JMP for three two-level continuous factors: endoprotease dosage, exoprotease dosage, and temperature. The leucine molar fractions (Leu%) of both Endo1 + Exo1 and Endo1 + Exo3 were reported.

Run	Endoprotease (mg EP/kg-Grist)	Exoprotease (mg EP/kg-Grist)	Temperature (°C)	Endo1 + Exo1 Leu%	Endo1 + Exo3 Leu%
1	0	0	52	7.54	7.68
2	0	0	52	7.66	7.57
3	100	0	52	10.38	10.72
4	0	100	52	7.47	7.86
5	100	100	52	10.17	12.90
6	100	100	52	10.04	12.99
7	0	0	63	7.28	7.42
8	100	0	63	7.97	8.50
9	100	0	63	8.24	8.61
10	0	100	63	7.27	7.65
11	0	100	63	7.32	7.68
12	100	100	63	9.50	11.41

**Table 2:** Parameter estimates and effect summary of Endo1 + Exo1 (left) and Endo1 + Exo3 (right).

Term	Parameter Estimate	*p* Value	Term	Parameter Estimate	*p* Value
Endo1(0, 100)	1.055625	0.00001	Endo1(0, 100)	1.6325	0.00000
Exo1(0, 100)	0.125625	0.02374	Exo3(0, 100)	0.695	0.00000
Temp(52, 63)	−0.421875	0.00028	Temp(52, 63)	−0.5125	0.00001
Endo1 × Exo1	0.154375	0.01201	Endo1 × Exo3	0.575	0.00001
Endo1 × Temp	−0.298125	0.00108	Endo1 × Temp	−0.4125	0.00003
Exo1 × Temp	0.226875	0.00303	Exo3 × Temp	0.08	0.01540
Endo1 × Exo1 × Temp	0.190625	0.00573	Endo1 × Exo3 × Temp	0.0775	0.01712

**Table 3:** Response-surface model of Endo1 dosage and temperature.

Run	Endo1 (mg EP/kg-Grist)	Temperature (°C)	Leu%	Predicted Leu%
1	83.5	52	10.18	10.18
2	0	52	7.67	7.67
3	39.5	57	9.32	9.36
4	39.5	57	9.39	9.36
5	100	59	9.58	9.58
6	100	59	9.59	9.58
7	0	62	7.50	7.50
8	63.5	63	7.97	7.97

**Table 4:** Parameter estimates in descending order of *p* value.

Term	Parameter Estimate	*p* Value
Endo1(0, 100)	0.8660797	0.0004
Temp(52, 63)	−0.762476	0.0006
Temp × Temp	−0.842294	0.0012
Endo1 × Temp	−0.515997	0.0021
Endo1 × Endo1	−0.375853	0.0056

## Data Availability

Not applicable.

## Supplementary Materials

The following supporting information can be downloaded at: https://www.mdpi.com/article/10.3390/molecules28114419/s1, Table S1: Selected volatiles after 2-day fermentation; Table S2: Selected volatiles after 4-day fermentation.

## Sample Availability

Samples of the compounds are not available from the authors.

## Appendix A

**Table A1:** Attenuation and alcohol by volume (ABV%) data of 4-day fermentation by *S. pastorianus*, *P. kluyveri*, *S. ludwigii*.

Yeast	Control		Endo1 + Exo3	
	Attenuation ^1^	ABV% ^2^	Attenuation	ABV%
*S. pastorianus*	60.9%	2.59 ± 0.05	61.2%	2.57 ± 0.02
*P. kluyveri*	8.2%	0.426 ± 0.002	7.6%	0.386 ± 0.002
*S. ludwigii*	8.3%	0.395 ± 0.002	7.7%	0.360 ± 0.004

^1^ Attenuation was calculated from original gravity, final gravity, and apparent attenuation. ^2^ ABV% was the mean of three independent fermentations, measured by HPLC-RI.

**Table A2:** Summary of selected volatile compounds and alcohol content after 4-day fermentation. Fermentation experiments were carried out in triplicate. Values are shown as peak areas. Values not connected with same letters are significantly different (*p* < 0.05).

Compounds		*Saccharomyces pastorianus*		*Pichia kluyveri*		*Saccharomycodes ludwigii*	
		Ctrl	Protease	Ctrl	Protease	Ctrl	Protease
Higher alcohol *	3-methylbutan-1-ol	27.18 ± 2.40 ^b^	33.48 ± 1.02 ^a^	10.71 ± 0.70 ^c^	4.47 ± 1.03 ^d^	14.18 ± 0.89 ^c^	26.47 ± 0.69 ^b^
	2-methylbutan-1-ol	7.58 ± 0.79 ^a^	7.39 ± 0.31 ^a^	2.56 ± 0.12 ^c^	4.27 ± 0.76 ^b^	1.39 ± 0.11 ^c^	1.52 ± 0.03 ^c^
	2-methylpropan-1-ol	2.33 ± 0.14 ^a^	1.61 ± 0.12 ^b^	1.44 ± 0.08 ^b^	1.51 ± 0.32 ^b^	0.39 ± 0.03 ^c^	0.39 ± 0.01 ^c^
	2-phenylethanol	36.61 ± 4.52 ^b^	24.62 ± 0.37 ^c^	13.61 ± 1.50 ^d^	4.02 ± 1.13 ^e^	40.94 ± 2.22 ^ab^	46.14 ± 0.96 ^a^
Higher acetate *	3-methylbutyl acetate	5.92 ± 0.65 ^b^	13.84 ± 1.96 ^b^	131.37 ± 16.81 ^a^	125.11 ± 23.32 ^a^	0.33 ± 0.05 ^b^	0.55 ± 0.06 ^b^
	2-methylbutyl acetate	3.34 ± 0.31 ^d^	6.40 ± 0.80 ^c^	28.07 ± 1.06 ^b^	34.93 ± 1.90 ^a^	0.21 ± 0.02 ^e^	0.31 ± 0.02 ^e^
	2-methylpropyl acetate	0.51 ± 0.04 ^c^	0.49 ± 0.07 ^c^	0.88 ± 0.13 ^b^	1.39 ± 0.27 ^a^	0.02 ±0.01 ^d^	0.02 ± 0.01 ^d^
	2-phenylethyl acetate	18.07 ± 2.48 ^c^	16.80 ± 1.12 ^c^	206.19 ± 18.52 ^a^	143.53 ± 14.92 ^b^	21.04 ± 1.40 ^c^	21.90 ± 1.37 ^c^
Medium-chain fatty acid ethyl esters	ethyl hexanoate	6.90 ± 0.60 ^a^	7.30 ± 1.11 ^a^	0.34 ± 0.15 ^b^	0.43 ± 0.10 ^b^	0.12 ± 0.02 ^b^	0.16 ± 0.02 ^b^
	ethyl octanoate	44.37 ± 10.82 ^b^	78.21 ± 14.92 ^a^	0.38 ± 0.18 ^c^	0.34 ± 0.08 ^c^	0.02 ± 0.04 ^c^	0.08 ± 0.07 ^c^
	ethyl decanoate	18.51 ± 1.97 ^b^	36.84 ± 9.80 ^a^	0.44 ± 0.06 ^c^	0.52 ± 0.20 ^c^	0.02 ± 0.04 ^c^	0.13 ± 0.05 ^c^
Other esters	ethyl acetate	5.66 ± 0.40 ^b^	7.93 ± 1.08 ^ab^	14.07 ± 5.09 ^a^	13.61 ± 4.13 ^a^	1.05 ± 0.04 ^b^	0.98 ± 0.04 ^b^
	3-methylpropanoate	0.028 ± 0.02 ^c^	0.03 ± 0.01 ^c^	2.2 ± 0.5 ^a^	1.0 ± 0.2 ^b^	0.0172 ± 0.0006 ^c^	0.019 ± 0.001 ^c^
Aldehyde off-flavor	3-methylbutanal	0.19 ± 0.06 ^b^	0.95 ± 0.27 ^a^	0.023 ± 0.004 ^b^	0.027 ± 0.002 ^b^	0.028 ± 0.002 ^b^	0.04 ± 0.01 ^b^
	2-methylbutanal	0.27 ± 0.09 ^b^	1.18 ± 0.05 ^a^	0.03 ± 0.01 ^c^	0.031 ± 0.04 ^c^	0.027 ± 0.002 ^c^	0.09 ± 0.01 ^c^
	2-phenylethanal	0.04 ± 0.01 ^b^	0.24 ± 0.04 ^a^	0.04 ± 0.02 ^b^	0.018 ± 0.003 ^b^	0.04 ± 0.01 ^b^	0.065 ± 0.003 ^b^
Alcohol by volume (ABV%)	-	2.59 ± 0.05	2.57 ± 0.02	0.426 ± 0.02	0.386 ± 0.002	0.395 ± 0.002	0.360 ± 0.004

* Compounds were validated by commercial analytical standards.
